# International law, public health, and the meanings of pharmaceuticalization

**DOI:** 10.1080/14636778.2014.951994

**Published:** 2014-09-18

**Authors:** Emilie Cloatre, Martyn Pickersgill

**Affiliations:** ^a^ Kent Law School, University of Kent, Canterbury, UK; ^b^ Centre for Population Health Sciences, University of Edinburgh, Edinburgh, UK

**Keywords:** access to medicines, drugs, patents, pharmaceuticalization, TRIPS

## Abstract

Recent social science scholarship has employed the term “pharmaceuticalization” in analyses of the production, circulation and use of drugs. In this paper, we seek to open up further discussion of the scope, limits and potential of this as an analytical device through consideration of the role of law and legal processes in directing pharmaceutical flows. To do so, we synthesize a range of empirical and conceptual work concerned with the relationships between access to medicines and intellectual property law. This paper suggests that alongside documenting the expansion or reduction in demand for particular drugs, analysts of pharmaceuticalization attend to the ways in which socio-legal developments change (or not) the identities of drugs, and the means through which they circulate and come to be used by states and citizens. Such scholarship has the potential to more precisely locate the biopolitical processes that shape international agendas and targets, form markets, and produce health.

## Introduction

We live, it seems, in an age of drugs. In spite of concerns that there is a crisis of innovation with the pharmaceutical industry (Hopkins *et al*. 2007), it is nevertheless clear that the use of chemical compounds to treat disease, enhance health, and practice recreation is increasing internationally: drugs “seem to be only growing in importance” (Sismondo 2004, 157). Indeed, as historian Greene notes, the expansion of access to pharmaceuticals “has become one of the most visible aspects of twenty-first century global health practices” (2011, 10). Some social scientists have characterized this emphasis on drugs within medicine, health and everyday life as a process of “pharmaceuticalization” (e.g. Abraham 2010; Biehl 2007; Fox and Ward 2008; Williams, Martin, and Gabe 2011).

In this analytic essay, we reflect on how pharmaceuticalization relates to the issue of access to medicines in “least developed countries.” We focus in particular on the nexus between access and intellectual property law, interweaving our own empirical findings with wider writings in this broad area of research (from science and technology studies (STS), sociology, anthropology, bioethics, and legal studies). In presenting such a synthesis, our paper represents neither a traditional literature review nor a pure research report. Instead, our contribution stands as an analytic essay that brings together various conceptual points and empirical findings into concert in order to prompt new conversations in STS and cognate disciplines about pharmaceuticalization (speaking to a broader project that we call the “social studies of law” (Cloatre and Pickersgill, forthcoming)).

Specifically, we want to urge for more social scientific attention to the role of “the legal” in constituting the social lives of drugs, and to assert that the place, role and impact of law and legal processes in directing pharmaceutical flows are complex, contingent, and multidirectional. Of course, as we will see later in the paper, these issues have already been a matter of concern for some scholars (perhaps most notably, for the sociologist John Abraham, who has produced a voluminous body of work on pharmaceutical regulation). Yet, international law and more socio-legal approaches to comprehending this are not the core features of STS and other writings on the place, role and impact of drugs in and on society.

This paper draws on examples of recent instances in which the complexity of the links between access and intellectual property law has been demonstrated. We attend especially to “TRIPS,” i.e. the World Trade Organization's Agreement on Trade-Related Aspects of Intellectual Property Rights. This is a controversial piece of legislation that is regarded as “plainly unjust” by many (Pogge 2008, 76), and is commonly associated with changing patterns of pharmaceutical consumption. Explorations of TRIPS are salient for broader considerations of pharmaceuticalization, especially if we are to be concerned with contexts outside of “the West.”^1^ Throughout, we understand law as a kind of “technology” in and of itself – a means by which material and semiotic change is leveraged (Lessig 1999; Pickersgill 2013; Yeung and Dixon-Woods 2010). What law “does” to drug markets and use is our key concern.

In what follows, we critically explore existing work on drugs and society, before interrogating and historicizing the (primarily legally oriented) debates around TRIPS and related access to medicines campaigns. We conclude with reflections on the import of this analysis for wider debates around pharmaceuticalization, and the use of interrogating the development and implementation of legal technologies for understanding this set of processes.

## Pharmaceuticalization: drugs and society

The concept of pharmaceuticalization has its origins in empirical social scientific analyses of the production, circulation and use of drugs (Petryna and Kleinman 2006), “objects whose social relations are more hidden” (Pollock 2008, 534) than other frequently studied actants within assemblages of health (such as physicians and biomedical technologies).^2^ More recently, medical sociologists Simon Williams, Paul Martin and Jonathan Gabe have defined pharmaceuticalization as “the translation or transformation of human conditions, capabilities and capacities into opportunities for pharmaceutical intervention” (Williams, Martin, and Gabe 2011, 711). Similarly, John Abraham refers to it as “the process by which social, behavioral or bodily conditions are treated, or deemed to be in need of treatment/intervention, with pharmaceuticals by doctors, patients, or both” (Abraham 2010, 290).

Following the seminal work of Conrad (2005), Abraham (2010) has argued that medicalization is a key mechanism by which pharmaceuticalization occurs, at the same time as deregulatory state policies and increased consumerism promote drug development and consumption. Particularly important examples of this can be found in the simultaneous expansion in the use of drugs aimed at treating mental ill-health, and the range of psychiatric disorders themselves that are recognized by different medical institutions (Lane 2009; Lee 1999; Scott 2006). The pharmaceutical industry, notes psychiatrist and historian David Healy, “now sell diseases rather than just drugs” (Healy 2006, 82).^3^ Recent scholarship has also brought to light the great extent to which psychopharmaceutical use may be operationalized as a form of “enhancement,” rather than “therapy” (Coveney, Williams, and Gabe 2011; Fox and Ward 2008; Hogle 2005; Martin *et al*. 2011; Williams, Martin, and Gabe 2011).

Research itself can be a powerful means through which health systems can become pharmaceuticalized (Dumit 2012), and clinical trials can act as a means of product promotion for drug companies (Petryna 2009). Indeed, it may be marketing departments that help to determine what trials are undertaken, and how the results will be disseminated (Healy 2006, 73). As Petryna (2009) shows, trials in low-income countries can act as platforms for the introduction of new drugs – which may sometimes be highly expensive, for pathological indicators (such as cholesterol) rather than “diseases” per se, and little more efficacious than existing treatments (see also Lakoff 2005; Petty and Heimer 2011). In such instances, it might be patients themselves who campaign for greater access to these pharmaceutical solutions to what may be symptoms of more pervasive problems in public health infrastructure.

Citizens, then, may actively ally themselves with what Adele Clarke and colleagues might refer to as the “Biomedical TechnoService Complex Inc.” (Clarke *et al*. 2010), and have consequently been active agents in pharmaceuticalization of their societies. For instance, as documented by Biehl (2007), in Brazil a policy of universal access to antiretroviral drugs (ARVs) was made possible through a novel alignment of activists, public sector institutions and the pharmaceutical industry itself. This had the effect of diverting public health priorities away from prevention, and toward the wide distribution of drugs. Medicalization, then, is not, a priori, necessary to support pharmaceuticalization. Instead, industry campaigns may work to enjoin consumers to recognize existing medical categories as necessarily needing more and better drugs to improve their condition (Abraham 2010). The alignment of patient and industry interests may be achieved in ways that have led to deep criticism of the industry; for instance, in regard to their direct sponsorship (and even creation) of patient/consumer groups (Lane 2009).

Macro-social trends in the production, circulation and consumption of drugs are powerfully governed by collective and individual responses to and conceptualizations of health, illness and the body. Pharmaceuticalization is intrinsically social: it is shaped by social and economic imperatives and structured by societal norms. These are in turn (co-)produced through a range of regulatory processes, including law per se. Accordingly, the role of governance and law in the mechanics of (de)pharmaceuticalization is gradually coming into sharper focus within social scientific and humanities scholarship (Carpenter and Tobbell 2011; Ecks and Basu 2009; Hayden 2007, 2011). Ideologies of deregulation have expanded the consumption of pharmaceuticals (Abraham 2010, 616);^4^ this is especially evident where patients and industry have collaborated through campaigns and litigation to improve access to drugs (Abraham 2010, 611; see also Biehl 2006, 2007). Indeed, regulators themselves have also played a role in increasing the number of drugs available on the market: in the USA, for instance, more lenient regulatory standards for cancer drugs have increased the focus of industry in this area (Davis and Abraham 2011). Contract research organizations (CROs) continue to examine the regulatory environment of prospective countries that might act as trial sites, assessing potential approval times for trials and whether it makes sense to market the drug there (Petryna 2009, 16). In what follows, we consider further some of the legislative aspects of drugs, paying particular attention to intellectual property.

## Making law, circulating drugs

When examining the legal context of drug use, it is difficult to miss the importance of patents. Granting a 20-year monopoly on specific medicines, patents define ownership and entitlements over a drug and uniquely position it within international markets. The patented drug becomes the first “face” that patients will associate with a specific pharmaceutical, often resulting in long-term fidelity. Consequently, patents shape the social nature of medicines, making them one of the most essential aspects of the regulation of pharmaceuticals.

The issue of how patents should be protected has always created very intense debates. Whilst they are formally a particular kind of ownership, and have the effect of creating a quasi-monopoly for a set period of time over pharmaceuticals, patents are also much more complex socio-legal tools that have the potential to embed elements of history, power relation, and conceptions of (in)justice. In addition, their deployment in practice follows material and social paths that make them unpredictable and messy actants (Cloatre 2013; Ecks and Basu 2009). The processes of social (dis)ordering that are generated by patents are therefore multiple; in their effects, they are sometimes subtle, sometimes profound.

The relationship between intellectual property and access to health has for a long time been studied through the lens of TRIPS and its impact. Patent law has traditionally been designed almost exclusively at the national level, but in 1995, TRIPS created new international standards in the field of intellectual property, and sought to introduce new minimum legal standards for the (strong) protection of IP into international trade. This ensured harmonization between WTO members with the aim of preventing infringements and granting the authority to exact injunctions and/or penalties where this occurs. TRIPS is associated with providing a strong legal basis for the protection of pharmaceutical patents granted in one jurisdiction in domains that had not previously respected these.

TRIPS has thus been considered to be particularly significant to countries that previously had no (or a very light) system of protection for patents on pharmaceuticals. In some cases, this was out of policy choice – in India, for example (Henderson 1997; Keayla 1999), where patent protection was offered for pharmaceutical *processes* but not *products*. This allowed for the reverse engineering of branded pharmaceuticals and the rise of a generics industry. Connecting capitalism to care, the Euro–American pharmaceutical industry argued that if their profits were harmed through generics, then R&D would decrease.^5^ Accordingly, strong patent protection was demanded, with concerns about infringement commonly articulated in public health terms (e.g. Peretz 1983, 262). In effect, the proliferation of generics was framed as having the potential to stall some of the machinery driving forward pharmaceuticalization (i.e. the innovation of new drugs) that were understood as being very good for global health (see also Ecks 2008, 165).

Following TRIPS, Euro–American industry concerns were translated into law. Soon after its signing, however, TRIPS was attacked for creating a danger for public health in developing countries. While generics had been widely portrayed as a threat to innovation (and, hence, ultimately, the pace and practice of pharmaceuticalization), the global expansion of drug patents that TRIPS generated became criticized for restricting access to medicines for the poorest (Picciotto 2002; Pogge 2008; Sell 2003). Critics commonly figure TRIPS as encouraging further pharmaceuticalization in high-income countries, while halting it in poorer nations.

Central to these discourses are the notions of “essential medicine,” specified by the WHO as the “minimum medicine needs for a basic health-care system, listing the most efficacious, safe and cost-effective medicines for priority conditions” (WHO 2011, 3). Such conditions are included “on the basis of current and estimated future public health relevance and potential for safe and cost-effective treatment” (WHO 2011, 3). Yet, distinguishing “essential” medicines remains problematic (Greene 2011, 10). Some suggest that since many essential medicines are off-patent, TRIPS will not impact negatively on health (Attaran 2004; Noehrenberg 2006). However, critics argue that it is precisely because drugs are off-patent (and hence cheaper) that they appear on the list (‘t Hoen 2010). Accordingly, we can begin to see the important role that patents can play in constituting the identity of drugs: in this case, whether or not medicines are “essential” (Cloatre 2008b).

TRIPS has thus activated a range of social, legal and health developments. Key questions pertaining to TRIPS include the following: with the extension of pharmaceutical patents to new spaces (increasing the monopoly of Big Pharma and decreasingly the market for generics), how would the price of drugs be affected? More pertinently, would this reduce access to medication for the poor and thus further increase global health inequalities? TRIPS therefore represents an important example of the ways in which legal tools can shape pharmaceutical consumption – and of how this is itself enrolled as a criterion for assessing both the need for and the effectiveness of law. The aforementioned account is also suggestive of the extent to which legal innovation animates new discussions about the management of health (e.g. the dialectic between concerns that pharmaceuticals are too seldom available, and worries that they are too often prioritized).^6^

## Translating global law

International law, as is the case for legal technologies more generally, can only acquire agency if enacted through local practice. Recent investigations into the social action that surrounds pharmaceutical patents and TRIPS in less-explored jurisdictions have demonstrated that the immediate authority, remit and relevance of international law are complex and contingent (Cloatre 2008a, 2008b, 2013). Whether countries were expected to create entirely new IP systems as a result of TRIPS (as is the case for many least developed countries, such as Djibouti, Cloatre 2008a, 2008b, 2013), or “simply” had to amend their national laws (for example Ghana; Cloatre 2013), the process of turning TRIPS into national law, and national law into new practices, is slow, complex and uncertain. In many contexts, the lack of local expertise, interest, or mobilization means that this is likely to take many years before any change in practices is noted. This needs to be accounted for in assessments of the role of law in directing pharmaceutical flows.

As we have discussed at length elsewhere (Cloatre 2013), there is a discrepancy between the imagined version of legal systems in which TRIPS is expected to be deployed, and the day-to-day micro-practices of law, health, and pharmaceutical markets in particular countries. In effect, the sociotechnical imaginaries (Flear and Pfister, forthcoming; Jasanoff and Kim 2009; Pickersgill 2011) projected by lawmakers may be distant from life as lived. More broadly, the enactment of law cannot be assumed through the existence of a small circle of intellectual property specialists; rather, the praxis of health professionals, public health experts, and perhaps also patients needs to be reshaped. Chains of association need to be forged, and in many places, in Africa and elsewhere, this is especially challenging given that pre-existing bridges between this diverse range of actors are rare. This complicates assumptions about the possibility of straightforwardly translating international law into specific nation states or localities – and, hence, challenges the idea that “global governance” really is global in nature and effects. Accordingly, the effect of TRIPS in these locations may be less direct or visible than reinforcing patents, and therefore (de)pharmaceuticalizing markets.

Our overall point is that a working pharmaceutical patent system is a complex sociotechnical network organized in diverse ways in different jurisdictions that depends on the ability of the system of import and marketing of medicines to respond to the requirements of patent law. The licensing of drugs, for instance, may be made to include a check on the patent status of particular drugs; if so, the post-market surveillance system will need to respond to this requirement: custom officers need to be aware of this particular set of regulatory limits on which specific versions of drugs may be allowed to enter the country, they need to have the material artifacts necessary for reporting, they need to recognize this as a feature of their everyday work that must not be ignored, there must be systems in place to ensure their compliance, and so on. Indeed, the deployment of such systems in practice may be problematic even in countries that have granted patents on pharmaceuticals for many years (Cloatre 2013), and is even more complex to create in the first place in states that have no pre-existing institutions. Consequently, in many places, law exists on paper, but not in practice. Without resources or expertise, and if it is seen to respond to the needs of others, elsewhere, rather than those experienced within the jurisdiction, legal tools are unlikely to be enacted. In what follows, we move from an examination of the kinds of debates TRIPS has stimulated to an exploration of specific practices of distribution and access to drugs, and the multidirectional effects that patents seem to have on the daily practices of health care.

## The shaping of drug markets^7^

In this section, we reflect further on the indirect role that patents can play in locations where the deployment of law is less visible or straightforward than the creators of TRIPS may have expected, examining how and why generic medicines can fail to become influential, even when patent law was formally implemented. The difficulties for generics to become central to public health in much of Africa, and in many least developed countries, are widespread (WHO and Direction de la Pharmacie, du Médicament et des Laboratoires 2004). Research we carried out in Ghana indicates, for example, that the efforts of the government to increase the use of generics in public hospitals are being held back by a routinized reliance on branded medicines (Cloatre 2013; see also Ghana Ministry of Health 2008). In particular, the entanglement of brands, as opposed to substances, in the practices of patients and health professionals, means that patented drugs are likely to occupy a strengthened position long after the rights granted by the patent have expired. The embedding of brands within doctors' prescriptions (Ecks and Basu 2009), themselves linked to factors ranging from ease and habit to proactive targeting by the pharmaceutical industry, is determinant in establishing which drugs get to patients. Furthermore, patents play a part in extending legal tools, which may, at times, mean that drugs will simply stop getting to patients. In some places, this will translate into a monopoly. In others, it means that the supply of cheap medicines to patients may be hindered by the fact that the market in cheap, generic medicines remains constrained and limited.

The example of Djibouti – a small country in the northeast of Africa that we have explored in more detail elsewhere (Cloatre 2008a, 2013) – is a good illustration of some of these complications, and it is worth discussing in a little more detail since it reveals the roles patents may play even in systems where they have, formally, no existence. Until recently, there was no patent law in Djibouti. In practical terms, this meant that any medicine could legally come into the country: the identities of drugs – whether there were branded, generic, or patented – were not simply uninteresting to, but in effect unrecognized by regulators who lacked any kind of legal framework through which to make visible the differences that TRIPS codified as so significant. Nonetheless, in spite of this a very narrow range of drugs was imported. In the private sector, which forms the biggest part of the pharmaceutical market, generic drugs were almost systematically excluded in practice. The absence of official law on pharmaceutical patents did not mean, therefore, that the drug market in Djibouti was unregulated; as recent approaches to regulation theories have highlighted, regulatory processes are often deployed aside from or instead of formal state regulations (Black 2002; Cloatre and Dingwall 2013). In this case, other agendas governed the importation of pharmaceuticals, in different and unexpected ways, revealing not only aspects of the direct influence of the global pharmaceutical industry that has been well documented, but also the internalizing and perpetuating of specific practices by local importers and health professionals. Harper, Rawal, and Subedi (2011) have demonstrated similarly complex links between the “unregulated” market of Nepal and the repeated dominance of branded medicines within it, although the processes that create this situation are slightly different in their example – and indeed, one of the features of these links between market and regulatory systems is their context-specific nature.

This suggests that the concerns of TRIPS critics and access to medicines campaigners that the agreement might further limit the opportunities of individuals in low-income countries to consume drugs do not hold everywhere – and in some contexts limitations are in place independently of the presence or absence of specific laws. Furthermore, concerns about TRIPS may perhaps need readjusting in other places. This is not to say that TRIPS, or that patents, are not highly problematic: instead, it suggests that the “problem” of patents may run deeper and be more subtle than one of “increased law/decreased access.”

## Access to medicines campaigns and pharmaceutical flows

The role of global regulation in shaping access to drugs, and processes of (de)pharmaceuticalization is also rendered more complex by the fact that the effects of any law are not only the creation of new rights, and new rules, but also wider social transformation (including mobilization and resistance). In the case of TRIPS, the processes that these new increased IP demands have generated have also provided international campaigns around access to medicines (such as those of Medecins Sans Frontieres) with significant impetus. Indeed, one of the effects of TRIPS has been to galvanize an opposition that has been effectively working at addressing some of the very issues that countries such as Djibouti and Ghana have faced.

In these resistant narratives, the processes of depharmaceuticalization that might be triggered or aggravated by TRIPS need to be addressed by increasing the flow of cheap medicines that are affordable for the poor. In turn, these campaigns themselves have attracted critique; for instance, some have questioned whether governments and NGOs today are too focused on the treatment of AIDS, and as such ignoring other diseases to the detriment of entire populations (Shiffman 2008). The wide range of programs created cannot be reviewed fully here, but a few points can be made to illustrate, again, the multidirectionality of the links between law and (de)pharmaceuticalization.

Again, the case of Djibouti is particularly telling, in that one of the biggest international programs set up in its health sector since TRIPS has been aimed at encouraging the import and use of generic medicines. This was targeted, mostly, through the development of dedicated “pharmacies communautaires” around the country, in which some cheap generics could be sold by bypassing the dominant import system described earlier. This means that in some locations in Djibouti, access to drugs may have paradoxically *increased* as a result of the mobilization generated by TRIPS.

The situation in Djibouti is illustrative rather than unique. In the past 15–20 years, campaigns for access to medicines have successfully exposed and transformed some of the injustices of the global pharmaceutical market. TRIPS can thus be understood to have played a significant, unanticipated and unwilling role in activating this mobilization (Sell 2003). Conceptually, this raises difficult questions in relation to how the role of the law may be approached. In particular, it suggests that linear understandings of legal processes, or indeed of processes of increased or decreased access to pharmaceuticals, might not account for the multifaceted ways in which global regulation affects local markets – or indeed, how we may wish to conceptualize the boundaries of what these legal regulations are and do (for example, the extent that resistance can be understood as “effects”). In regard to understandings of pharmaceuticalization, we can see that the movements of particular drugs are shaped by the ambiguous effects of specific “global” and “local” events.

In addition, the transformations that followed the global mobilization against IP and for access are played out in ways that are not only about processes of (de)pharmaceuticalization, but also about changes in the *type* of pharmaceuticals that are the focus of health priorities. One effect of the debates on IP, for example, has been to increase awareness of the difficulty in getting access to patented (“modern”) medicines. In particular, international attention has most heavily focused, in response to TRIPS, on funding and sustaining access to the few drugs on the WHO's essential medicines list which are under patent (and hence expensive) – primarily, ARVs for HIV/AIDS (MSF/WHO/UNAIDS Secretariat 2003). As the cost of ARVs and the impact that patenting rules had on them became more publicized, extensive campaigning focusing on access emerged globally, and funding for these has steadily increased since the mid-1990s. As a result, access programs for ARVs have been implemented in many countries. The debate has subsequently broadened to question the prioritization of “modern drugs” over “basic medication.” Processes of shifting attention to drugs in general also involve decisions over which drugs should be prioritized. Such discourses underscore how “AIDS medicine is socially as well as pharmacologically active in that it occasions reflections on social relations and distinctions” (Reynolds Whyte *et al*. 2006, 241).

Alongside ARVs, particular attention has been paid to TB and malaria, two diseases for which the issue of innovation and access to new medicines is acute. Again, one question that has arisen in response to the heed paid to TB and malaria is the extent to which this has meant that attention has shifted from issues of generalized access to pharmaceuticals as a whole, to issues of access to certain type of medicines. Moreover, the progressive reshaping of drug markets through access programs has raised new questions about dependency and (de)location of the making of drugs that, once more, complicate analytic comprehension of the social, legal and political dimensions of global flows of medicines. For example, the emergence of new regulation for the making of medicines for globally funded programs, and in particular the WHO pre-qualification program, is increasingly constraining and delimiting which drugs can or cannot be enrolled into local pharmaceutical markets. Access to particular medicines therefore becomes conditioned on the enrollment of particular places of production (not only of medicines, but also of regulatory knowledge, where technicalities as explored by Riles (2011) become the key to ordering and socio-legal processes). Access to a particular drug is contingent not only on the incorporation of patients in particular medical regimes (as explored by Ecks 2008 in the case of providing free access to Glivec), but also on the emplacement of drugs in particular systems of regulatory knowledge.

In sum, critiques have been voiced against a seemingly increased attention to treatment, and pharmaceutical treatment in particular, through access to medicines campaigns, to the detriment of prevention (Biehl 2007). At the same time, the shifts in focus between “new substances” and “basic medication,” and between different regulatory systems (including emerging ones that regulate donations and access programs), order in different ways the types of drugs that become mobilized, sections of industry that become (dis)empowered, and the position of different localities (from laboratories to hospitals) that are understood as valid spaces to produce better health.

## Discussion

Internationally, shifts can be discerned in regard to understanding health as a commodity – one that can be secured primarily through access to drugs (as opposed to other measures, including infrastructure). Such discourses focus particularly on the plights of citizens in low-income states, where images of the deserving poor are juxtaposed with descriptions of greedy Euro–American pharmaceutical firms. TRIPS is criticized as a legal tool that has the potential to dismantle the machinery which might best enable medicines to freely circulate, and efforts have been made (e.g. the Doha Declaration) to limit this possibility. Other actors, however, critique the choices made by the WHO, NGOs and nations in regard to which drugs are deemed essential for citizens to access. What is foregrounded in these narratives is the importance of medicines for some diseases over or relative to others. This may take the form of concerns that key diseases are being neglected (e.g. malaria) in general, or specifically in comparison to others (in particular, HIV/AIDS). Even more voices can be heard from the pharmaceutical industry. They speak of drugs as, again, being essential to health, and regard the saturation of markets with generics as worrying since it might ultimately stall or halt the engines of pharmaceuticalization (e.g. by reducing industry profits, and hence their capacity to innovate). Just like those actors and associations involved with access to medicines campaigns, the pharmaceutical industry deems more, and better, drugs to be the key to health. This notion is concretized in the list of essential medicines maintained and regularly revised by the WHO, and is at once sustained by and a contributor to global processes of pharmaceuticalization. We do not make this observation in order to suggest that drugs should not play a (perhaps key) role in public health. Rather, we aim to underscore the sometimes surprising alignments of interests and sociotechnical imaginaries that are constitutive of and produced through processes of de/pharmaceuticalization.

Dialogue and debate around drugs in low-income countries are thus marked by actual and imagined processes of de/pharmaceuticalization; as a whole, the discursive space within which nations, NGOs, and international agencies operate is marked by ambivalence about the nature and (possible) effects of law, the meaning of health, and the propriety of particular kinds of intervention. Legal processes play out across a mixture of porous boundaries and rigid networks, in which “local” situations can have major import for the instantiation of “global” regimes. These barely contained complexities explode when we attend to particular case studies. In narrating the specific features of the Djibouti drug market, we have signaled the “excess” of power that patents can have, and the ways in which they inform perceptions of the materiality of the products to which they adhere (cf. Barry 2005). Patents in Europe and elsewhere grant certain drugs a monopoly in those nations, which might translate into a kind of prestige or desirability elsewhere when professionals “brought up” with those brands in the regulated zone move to jurisdictions without IP law. This enables a de facto system of regulation wherein patents have power even where they do not officially exist, profoundly shaping drug markets and limiting the extent to which public health in Africa can become de/pharmaceuticalized. Thus, like Biehl (2007), we assert that policy-makers and patients can be subject to the enchantment of particular kinds of drugs; yet, in contrast to his findings, the appeal of particular chemical compounds does not necessarily result from their perceived necessity or novelty, nor is it constituted through new arrangements between citizens and the state. In effect, pharmaceutical use in Djibouti or in Ghana following TRIPS looks remarkably like “business as usual” for pharmacists and those with whom they trade (though, as we have indicated, some developments have occurred). This is largely because the relationship between law and daily practices is messy, uncertain and reciprocally constitutive.

The multifaceted nature of the empirical reality we have sought to capture through this analysis encourages, we believe, further debate about the scope, limits and potential of the notion of “pharmaceuticalization” as a lens through which to visualize the social life of drugs. In our account, particular axes of debate and action can be located that have been disciplined through characterizing them as processes of de/pharmaceuticalization. Yet, we have clearly not produced a straightforward narrative of a uniform increase (or decrease) in drug consumption. Rather, we have analyzed the extent to which past, current, and future laws both inscribe and enact particular visions of health and the means of ensuring it, and how these in turn impact on what kinds of pharmaceuticals are used, where, and for what reasons. Undoubtedly, “pharmaceuticalization” seems an important concept with which to approach research on drugs and direct our analytical gaze (and the existing literature in this area is innovative and insightful). Arguably less desirable, however, would be research that aims *solely* to document a rise (or fall) in pharmaceutical production and consumption – and which, in the process, elides the contradictions and complexities that characterize and constitute these patterns of use. It is precisely these that are necessary to attend to in order to map the textured terrain within which drugs travel.^8^

## Conclusion

The emphasis of pharmaceuticals within current health systems “raises vital questions about public health priorities and their financing, and the role of equity in the human right to health” (Petryna 2009, 45). Our synthesis of a range of empirical and conceptual work on law, drug markets, and access to medicines campaigns has enabled us to produce a narrative that elucidates some of the complexities that “the legal” introduces to understandings of pharmaceuticalization. In particular, our contribution is to show how international law is being used to shape these processes, but that its deployment has sometimes contingent and limited effects – and that these are in turn structured by older forms of law through their interactions with drugs. Our essay has thus documented the importance of both law and legal processes to scholarly engagements with pharmaceuticalization, while showing too that “the legal” simultaneously channels pharmaceutical flows in different ways and that it cannot be readily assumed without attention to specific cases. Our (admittedly selective) attention to the role of law and governance in processes of pharmaceuticalization also enjoins some reflection on the term itself. We suggest that this provides an important organizing framework for studies of the production, circulation and use of drugs, but wish to flag that (as with concepts such as medicalization and geneticization) analysts who restrict their gaze to an axis of de/pharmaceuticalization potentially obscure the complexity of the biopolitical matrix within which pharmaceuticals come to have a social life.

## Notes

1. See Bell and Figert (2012), but also Williams, Gabe, and Martin (2012).

2. As Williams, Gabe, and Martin (2012) remind us, in spite of the relative novelty of the term “pharmaceuticalization,” social scientific engagements with drugs have been evident in the literature for at least 30 years.

3. Importantly, the pharmaceutical industry also provides expertise: “companies are producers not only of pills but also knowledge about their safety and efficacy, and their gifts to doctors of travel to conferences and workshops provide access to the latest expertise” (Lakoff 2005, 140).

4. See also Abraham (2007) and references therein.

5. It is perhaps worth noting that some social scientists have argued that the costs of pharmaceutical innovation are not as high as international manufacturers often claim (Light and Warburton 2011).

6. The formation of TRIPS is also an illustration of the complex ways in which so-called global systems are constituted, and of the mobility of particular kinds of law.

7. A fuller account of the patent situation in Djibouti is presented in Cloatre (2013).

8. There is also a danger that the focus on pharmaceuticalization as a contemporary concern will also mask the historical context of drug marketing and use; the pervasive place of the pharmaceutical industry in medicine and society is nothing new (Greene and Podolsky 2009) and hence the challenge for scholars is to more precisely locate which shifts represent new and important breaks and which are intensifications of processes that have documented antecedents.
